# Discharge Energy Versus Exposure Time in Atmospheric-Pressure Air Plasma Surface Treatment of Polyimide and Polyamide 6 Films

**DOI:** 10.3390/polym17101394

**Published:** 2025-05-19

**Authors:** Iustina Hatescu, Cătălin Borcia, Roxana Ciobanu, Gabriela Borcia

**Affiliations:** Iasi Plasma Advanced Research Center (IPARC), Faculty of Physics, Alexandru Ioan Cuza University, Blvd. Carol I No. 11, 700506 Iasi, Romania; tinahhatescu@gmail.com (I.H.);

**Keywords:** polyimide, polyamide 6, atmospheric pressure plasma, wettability, adhesion, oxidation

## Abstract

Polyimide (PI) and polyamide 6 (PA6) films are treated under exposure times of 0.5 s and 1.0 s, and energy levels of 1.5, 2.0, and 2.3 mJ/pulse. PI exhibits the most substantial improvement in wettability and adhesion-related properties compared to PA6 and other studied polar polymers. The threshold level for stable surface modification is reduced, achieving a minimum water contact angle of 45°. The stability is markedly enhanced, with aged PI surfaces showing a 40% relative increase in adhesion work compared to untreated samples. The oxygen content on the PI surface reaches 22 at. %, surpassing the maximum of 18 at. % O observed for PA6. The surface roughness of PI increases by approximately a factor of 2, while PA6 shows an average increase of only 25%, attributed to higher ablation rates in the amorphous phase compared to the crystalline phase. The degree of surface modification achieved with [1.0 s; 1.5 mJ] treatment parameters is comparable to that with [0.5 s; 2.0 mJ], demonstrating that higher discharge energy can effectively shorten the required exposure time. This plasma treatment, even at very short exposure times, enables significant enhancement of the surface properties of PI, typically characterized by high chemical stability.

## 1. Introduction

The surface of polymers, materials with unparalleled range of bulk characteristics [1], is typically intrinsically hydrophobic and chemically inert, which is not suitable for applications that require adequate wettability, adhesion, lubrication, tribology, bondability, or biocompatibility. Specific technologies are necessary to adjust the polymer surface properties.

Low-temperature plasma is a distinct method for processing sensitive polymer surfaces. It provides rapid processing, high production efficiency, low energy consumption, and minimal impact on bulk properties, such as mechanical characteristics, as it affects only a limited number of subsurface layers [2,3,4,5,6,7,8,9,10]. Among plasma processing techniques, atmospheric-pressure plasma (APP) eliminates the technological costs associated with conventional low-pressure plasma equipment while significantly reducing the generation of harmful by-products in both gaseous and liquid phases. APP operating in air represents the most promising technological solution [6,7,11,12,13].

APP sources for polymer treatment are designed to operate far from thermal equilibrium and should generate large-scale plasmas with a uniform distribution of active species. In this context, dielectric barrier discharge (DBD) provides a practical solution. Using short voltage pulses with a high rise time helps increase the interelectrode gap, ensuring better discharge stability, as well as reaching a high degree of non-equilibrium, with enhanced vibrational excitation of gas molecules [11].

This study expands upon previous research on the surface modification of polymers using an APP reactor in air. The plasma is generated using a DBD of the random filamentary type, less sensitive to minor changes in electrode configuration or small variations in the amplitude or repetition frequency of the applied voltage [14,15]. The enhanced electrode configuration provides better control over reproducible discharge conditions, while the uniform sweeping of the sample through short-lived random microdischarges ensures rapid and uniform treatment.

Previous studies focused on nonpolar polymers, which lack functional groups and exhibit high hydrophobicity. Polyolefins, such as polyethylenes (PEs) and polypropylene (PP), as well as polystyrene (PS), enabled the evaluation of treatment efficiency in terms of adhesion enhancement and oxygen uptake following short plasma exposure [14].

The study was extended to polar polymers. Polyethylene terephthalate (PET), polyetheretherketone (PEEK), and polymethyl methacrylate (PMMA) enabled the evaluation of plasma-induced additional oxidation in materials with varying oxygen contents. Due to the distinct behaviour of PMMA, which has a limited extent of modification compared to other polymers, the findings highlighted the importance of assessing each specific combination of plasma, polymer, and treatment parameters [15].

In this study, different polymer classes, such as polyimide and polyamide, are selected, and the range of treatment parameters is extended. The surface properties are analyzed using contact angle measurements (CA), X-ray photoelectron spectroscopy (XPS), and atomic force microscopy (AFM). X-ray diffraction (XRD) is used to examine the amorphous/crystalline structure. The stability of surface properties is assessed by measuring the water contact angle (WCA) over a two-week period following treatment.

Polyimides (PIs) are a class of aromatic heterocyclic high-performance polymers, with excellent resistance to high temperatures, mechanical stress, solvents, and chemical attacks, along with thermal, electrical, and sound insulation properties, long-term durability, and more [16,17,18]. PIs are widely used in key technological fields such as aerospace engineering and biomedical devices. They are particularly well-suited for flexible devices, especially in flexible electronics, where strong bonding between PI and other materials is critical [19,20,21,22,23,24].

Polyamides (PAs) are an important class of engineering thermoplastics known for their durability, high tensile strengths, puncture resistance, resistance to corrosion and chemicals, thermoformability, flexibility, transparency, good barrier characteristics, and cost-effectiveness, with broad applicability across industries [25,26,27].

Relatively few studies have investigated the effects of plasma exposure on PIs, and even fewer have focused on APP, when compared to PAs.

Table 1 provides a schematic overview of the APP treatment parameters and results for PIs. The literature reflects a sustained interest in enhancing the adhesion properties of PI through plasma technology. Research has extended to various PI forms, including films, tapes, sheets, membranes, and powders [21,28,29,30,31]. For instance, Gerulis et al. employed APP to replace surfactants and improve the dispersibility of PI powders in solution [31]. Recent studies highlight ongoing efforts to develop APP sources in diverse configurations, such as plasma jets, DBDs, and microwave APP sources, with a particular focus on achieving high spatial uniformity over large surface areas [21,32,33,34].

Suzuki et al. report an impressively rapid modification of PI using APP, achieving effective treatment within just 20 ms of exposure and maintaining good spatial uniformity over a 20 cm length [32]. In this setup, Ar gas is necessary to sustain a stable and uniform plasma. The WCA of plasma-treated PI increases rapidly with the distance between the sample and the plasma exposure slot, with the lowest WCA observed at a minimal separation of 0.5 mm. Prolonged exposure leads to rapid ashing of the polymer, while the long-term stability of the treatment remains unassessed.

Several studies have reported high levels of wettability in plasma-treated PI, in some cases reaching superhydrophilic characteristics [28,32,33,39,41]. This effect is generally attributed to strong morphological changes induced by plasma treatment, such as the formation of visible features like particles or stripe-like patterns. The evenly distributed nanoscale particles may disrupt the surface tension of water droplets, allowing them to spread easily and resulting in superhydrophilicity [33,41]. Similarly, many studies report rather severe morphological modifications, including etching effects [19,28,29,33,34,41]. When evaluated over time, a significant recovery of the wettability towards its initial level is consistently observed.

Table 1 also includes selected references on the APP treatment of PAs, with a focus on recent publications and studies emphasizing short treatment durations. Additional studies are reviewed in [26], where most reported minimum WCAs range between 20° and 40°, with longer treatment times generally leading to improved wettability.

A reference to subsecond treatment can be found in [44], where PA12 films were treated using microwave APP in argon, with oxygen and nitrogen admixtures to promote functionalization. The system employs a complex plasma source that generates significant heat, requiring both the source and the sample to be cooled during operation. The plasma-induced hydrophilic surface rapidly recovers after exposure.

In [45], PA6 treated with volume dielectric barrier discharge (VDBD) and diffuse coplanar surface barrier discharge (DCSBD) for exposure times of up to 2 s exhibited a strong ageing effect. Longer treatment times were necessary for improved stability. Both DBD configurations required very short distances between the electrodes, up to a maximum of 1 mm, limiting the treatment to thin, uniform films.

In [46], a DBD at atmospheric pressure in air reduced the contact angle on PA66 foils from 64° to approximately 30° after 0.5 s of plasma treatment, with minimal recovery within the first 4 days post-treatment. However, the ageing trend did not stabilize, and longer treatment durations resulted in greater stability compared to shorter ones. The DBD setup features a large metal HV electrode, requiring a very short distance between electrodes to ignite the discharge, along with a robust HV supply to generate high power to sustain the discharge.

As summarized in Table 1, many studies report plasma exposure times ranging from seconds to several minutes, with only a few examining subsecond exposure. The stability of the initial wettability of plasma-treated polymers is not always assessed. For nitrogen-containing polymers, the apparent increase in nitrogen content, expressed as the N/C ratio rather than the N1s relative percentage, could reflect surface oxidation, which lowers the relative carbon percentage, thereby artificially increasing the N/C ratio. In some studies using commercial APP generators, key experimental details, such as electrical parameters, setup dimensions, and actual treatment duration, are not fully disclosed. Unspecified treatment durations generally involve multiple passes of the sample, meaning the actual plasma operation time may be longer.

Studying short APP treatment on PI and PA6 is valuable for assessing the reactor’s performance in improving wettability and adhesion-related properties across different polymer classes, with a focus on post-treatment stability. The lower oxygen content in PI and PA6, compared to previously studied polar polymers in a similar DBD setup [15], enables the evaluation of the factors influencing additional surface oxidation, considering both their initial oxygen content and the stability of their chemical structures. PI and PA6, which contain amide-like groups, allow for the examination of high-polarity structures with stronger intermolecular forces and carbon–nitrogen bonds under APP exposure.

It is particularly interesting to investigate the short APP exposure outcomes and post-treatment stability in the unique chemical structure of PI, which may offer enhanced chemical stability compared to other polymers that have been more extensively studied.

APP in ambient air provides uniform modification across exposed areas of about 15 cm × 15 cm, with a rapid effect occurring within the first 0.5 s of exposure. Increasing the energy applied to the discharge further optimizes the degree of surface modification. The primary mechanism is functionalization, with minimal post-treatment hydrophobic recovery. PI shows the most significant improvement in surface properties compared to other studied polar polymers under similar treatment conditions.

These findings validate the APP technology developed in our laboratory for the controlled activation and modification of polymer surfaces. The testing covers key classes of commercially important materials, including commodity, industrial, and high-performance polymers. Surface properties demonstrate the achievable limits of surface modification in terms of hydrophilicity, adhesion, surface polarity, and oxygen uptake within the current experimental setup.

These results also contribute to the broader field of polymer-based materials, particularly the less-studied polyimides and related composites, where adjusting adhesion-related properties is essential, while preserving bulk properties and surface stability to ensure long-term operational stability.

## 2. Materials and Methods

**Figure 1:**
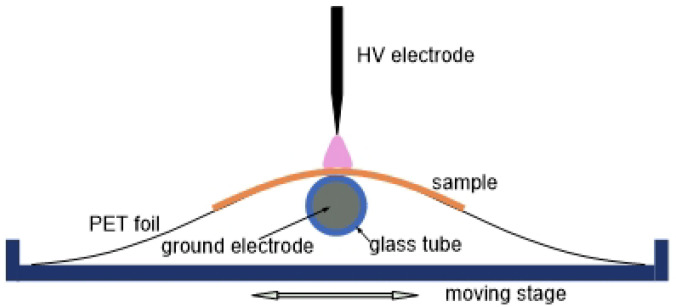
Schematic diagram of the DBD configuration.

The plasma used for surface treatment is generated by a custom-designed and -built reactor operating in air at atmospheric pressure, based on a DBD. The DBD configuration, which produces APP, is similar to that used in previous studies [14,15]. In this work, the asymmetric two-electrode system has been modified, as schematically shown in Figure 1, to enable enhanced control over the discharge regime and operational parameters.

The HV electrode comprises a stainless steel blade (20 cm long, 2 mm thick) with rounded edges, designed to prevent localized field enhancement and ensure a uniform discharge. The ground electrode consists of a stainless steel bar (22 cm long, 4 mm in diameter) encased in a glass tube (inner diameter 4.2 mm, outer diameter 6.2 mm), which acts as the dielectric barrier. A dielectric foil (0.2 mm thick PET polymer film, 22 cm wide, 25 cm long) is placed over the ground electrode, serving as the substrate support for the samples to be treated.

The compact electrode design allows for precise alignment due to the small radii of both electrodes. The smooth surface of the glass tube and adjustable positioning of the HV electrode facilitate accurate control of the interelectrode gap. The gap can be extended up to 2–3 mm, allowing treatment of thicker or porous materials, such as wovens, etc. The narrow discharge width (2 mm) helps to prevent overexposure of the sample, eliminating the need for a large planar setup spanning dimensions of around 10 cm.

During treatment, a motor stage moves the PET foil substrate, enabling the samples to pass through the discharge region at a linear speed ranging from 0.3 to 3 cm/s. This setup enables control of very short treatment durations, down to fractions of a second.

The discharge is produced by an electric circuit that generates HV pulses with a duration of 40 μs, a repetition frequency of 900 Hz, and a variable amplitude ranging from 10 to 12 kV, resulting in peak current intensities of 6 to 8 A.

APP is generated in a filamentary mode, characterized by continuous electrical breakdown through numerous microdischarges. The plasma appears as a continuous “curtain” with a volume of approximately 20 cm × 2 mm × 2 mm. The short-lived filaments are randomly distributed within the interelectrode zone, and the continuous movement of the sample ensures uniform treatment of test surfaces up to approximately 15 cm × 15 cm in size. The uniformity of the treatment was evaluated using contact angle measurements at multiple sites across the larger treated surface. The variation in measured values was less than 2° across the entire exposed area.

The actual plasma exposure time is calculated based on the linear speed of the sample under the discharge and the discharge width on the sample.

**Figure 2:**
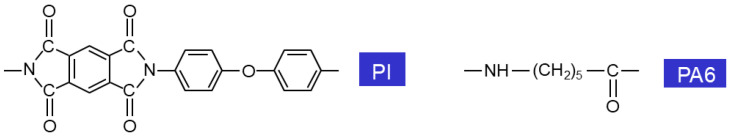
Chemical structure of the repeat units of PI and PA6.

The experiments are carried out on polyimide (PI) and polyamide 6 (PA6) commercial polymer films (0.05 mm thickness) (Goodfellow Ltd., Cambridge, UK) (Figure 2). PI and PA6 were treated with plasma under varying exposure times and electrical discharge parameters. The exposure durations were chosen to enable comparisons with previously studied nonpolar and polar polymers [14,15]. Plasma exposure durations of 0.5 s and 1.0 s were tested at an energy of 1.5 mJ/DBD pulse. Then, the exposure was maintained at 0.5 s while adjusting the high voltage (HV) to achieve three distinct energy levels: 1.5, 2.0, and 2.3 mJ/DBD pulse.

The crystalline structure of the polymer films was investigated by X-ray diffraction (XRD) using a Shimadzu LabX D6000 X-ray diffractometer (Shimadzu, Kyoto, Japan), with a Cu-K_α_ X-ray source (λ = 1.54059 Å) in a Bragg/Brentano configuration. The samples were scanned in the 2θ = 10–80° range, at a 4°/min scanning rate and 2° grazing incidence. The diffraction patterns show peaks, associated with the diffraction in the crystalline phase, superimposed on an amorphous halo. These patterns are fitted with mixed Gaussian/Lorentzian functions, with a mixing ratio > 0.8 and linear-type background subtraction. The degree of crystallinity X_c_ calculated from the ratios of the areas under the crystalline peaks A_c_ and the amorphous halo A_a_, per [50], is

X_c_ = A_c_/(A_c_ + A_a_) (1)

The contact angle measurement (CAM) was carried out by the sessile drop technique using an automated system to store the drop images via an Optika 4083.B5 digital camera (Optika, Ponteranica, Italy) with PC-based control, acquisition, and data processing. The measurements were conducted at room temperature, ranging between 19 and 21 °C. The values of the static CA presented are the averages of at least 10 measured values obtained on the imaged sessile liquid drop profile, with a drop size of 1 μL.

The water adhesion work on treated surfaces is calculated as

W_a_ = γ_L_(1 + cos θ), (2)

where θ is the contact angle, and γ_L_ is the surface tension of the test liquid, water (W) or formamide (F), presented in Table 2.

The relative increase in the adhesion work, defined as

ΔW_a_/W_a_ = (W_a(treated)_ − W_a(untreated)_)/W_a(untreated)_ × 100%, (3)

is also used as a control parameter for the plasma-induced effect.

The values in Table 2 are used to calculate the surface energy (γ_S_) and its polar (γ_S_^p^) and dispersive (γ_S_^d^) components, using the Owens, Wendt, Rabel, and Kaelble (OWRK) model [51,52]. The total surface energy of the polymer sample is

γ_S_ = γ_S_^d^ + γ_S_^p^ (4)

and the surface polarity is defined as

γ_S_^p^/γ_S_ = γ_S_^p^/(γ_S_^d^ + γ_S_^p^). (5)

The contact angle of water (WCA) was used to monitor the surface’s ageing post-treatment. The post-treatment measurement of WCA was conducted on material strips (~8 cm × 0.5 cm) cut from a larger piece that had been exposed to plasma. The strips were then stored in sealed boxes, in a dry and cool room, throughout the ageing process, and WCA was measured at various intervals up to 14 days after plasma exposure to assess the surface’s tendency to reduce its surface energy.

The surface chemical analysis was performed by XPS, with the XPS spectra recorded on a PHI VersaProbe 5000 spectrometer (ULVAC-PHI, Kanagawa, Japan) using the Mg-K_α_ line (h*ν* = 1253.6 eV), at a 45° take-off angle and 20 eV pass energy. The peak envelopes were curve-fitted using PHI MultiPak software (ver. 9.6, Ulvac-PHI, Inc., Chikasaki, Japan), employing mixed Gauss/Lorentz component profiles with a mixing ratio > 0.8 and linear-type background subtraction.

The surface morphology of the samples was analyzed by atomic force microscopy (AFM) using a Scanning Probe Microscope (Solver PRO from NT-MDT, Moscow, Russia) in non-contact mode. In order to allow for quantitative comparison of the surface roughness across the investigated samples, all images were taken during one imaging session using the same cantilever (NSC21 from MikroMasch, Sofia, Bulgaria) with a typical curvature radius of 10 nm, nominal spring constant of 19.1 N/m, and free resonant frequency of 227.9 kHz), and the same laser position. Microscope control, data acquisition, and image analysis sere performed using Nova software from NT-MDT, version 1.0.26.1443 from NT-MDT. The surface morphology was characterized by means of texture parameters, such as arithmetic mean roughness S_a_, root mean roughness S_q_, and maximum roughness S_y_.

## 3. Results and Discussion

### 3.1. Amourphous–Crystalline Structure

**Figure 3:**
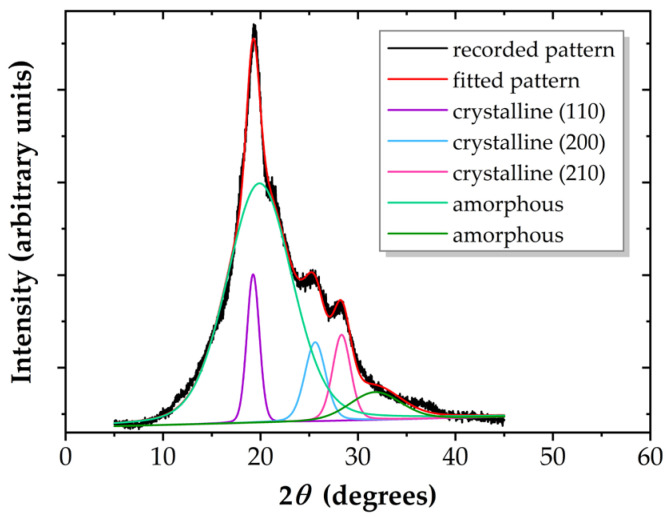
Curve-fitted diffractogram for PI.

The two polymers have a different amorphous–crystalline structure. The diffractograms show a single diffuse signal for PA6, which demonstrates its completely amorphous structure. For PI, three narrow signals corresponding to the crystalline phase are highlighted, located at 2θ = 19.24°, 25.62°, and 28.32°, as shown in Figure 3.

Fitting the diffractogram, in correlation with data from the literature [53,54,55,56], leads to a crystallinity index X_c_ = 0.28, which indicates the semicrystalline nature of PI. This value is comparable to those calculated for polyethylenes (PEs) [14], but corresponds to a moderate crystalline character compared to the previously studied highly crystalline PET polymer, which has X_c_ = 0.67 [15].

Since plasma may influence the surrounding amorphous macromolecular matrix at a higher rate than the embedded crystallites [42,46], variations in the amorphous–crystalline structure of these polymers could lead to different treatment effects.

The diffractograms do not change upon plasma exposure, since plasma modifies only a superficial layer.

### 3.2. Surface Wettability, Adhesion, and Polarity

The CAM and subsequent calculations indicate overall that APP exposure enhances wetting and adhesion-related properties, with PI showing the most significant improvement, compared to PA6 and other studied polar polymers [15].

PI and PA6 have hydrophilic surfaces, with similar WCA, in a narrow range of 75–80°, and undergo higher wettability upon plasma exposure (Table 3). The WCA values measured immediately after treatment also fall within a similarly narrow interval. WCA decreases to ~35–42° for PI and ~39–45° for PA6, showing dependence on both treatment parameters, i.e., exposure duration and DBD applied energy.

Measurements taken from different strips of material cut from the larger piece (approximately 15 cm × 15 cm) showed consistent values within the error limits reported in Table 3 (maximum ± 1.1°). This confirms the uniformity of the surface treatment, aligning with observations from previous studies on other polymers [14,15].

The rate of surface modification is highest during the first 0.5 s of treatment, with surface parameters continuing to change at a slower rate as exposure time increases. For an energy of 1.5 mJ/DBD pulse, WCA decreases noticeably when treatment time is doubled, with a reduction of 6–7° compared to the 0.5 s treatment. For a constant plasma exposure duration of 0.5 s, the WCA decreases as the DBD energy increases. The level of surface wettability is nearly identical (within error margins) for the treatment parameters [1.0 s; 1.5 mJ] and [0.5 s; 2.0 mJ], indicating that higher discharge energy can achieve effective surface modification with shorter exposure times.

**Figure 4:**
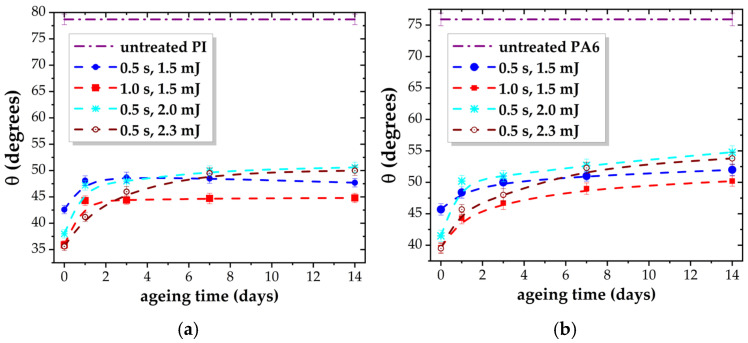
Variation in WCA vs. ageing time for plasma-treated (**a**) PI and (**b**) PA6 as a function of exposure time and DBD applied energy.

The difference between the WCA values for PI and PA6 is not large, but it is distinct, especially as the measurements are affected by small errors (±0.8–1°), as also marked with error bars in Figure 4. It was found that the modification of PI is stronger than that of PA6, although PI has a dominantly cyclic and aromatic structure and an initial oxygen content higher that of PA6.

This behaviour is consistent with studies by other groups, which have shown that plasma exposure of PI can lead to accentuated hydrophile characteristics, although after longer exposure times [20,28,33,39]. It also aligns with our previous results, demonstrating that under the current DBD configuration, the modification of the aromatic macromolecular structure is more pronounced compared to that of the aliphatic structure under the same treatment conditions [14,57].

The ageing-related evolution of hydrophilicity, illustrated in Figure 4, shows differences for the two polymers. The most significant reduction in wettability occurs within the first 2–3 days after treatment for both materials. However, PI shows a clear stabilization of properties after this initial period, whereas PA6 continues to exhibit gradual changes in wettability for at least two weeks post-treatment.

After ageing, the measured values remain within a narrow range of 45–50° for PI and 50–55° for PA6, indicating the highest susceptibility of PI to plasma modification. In the current DBD setup, it lowers the threshold for wettability modification of polar polymers to a minimum WCA of 45°, compared to 50° reported in our previous studies [15].

Table 4 presents the parameters associated with the adhesion-related surface properties, specifically water adhesion work (W_a_), calculated using Equation (2), and its relative variation (ΔW_a_/W_a_), calculated using Equation (3).

W_a_ on the treated PI and PA6 surfaces reaches levels comparable to those observed in previously studied polar polymers [15], confirming the efficiency of the APP treatment across different macromolecular structures. In this case, the stability of adhesion-related properties is significantly improved.

ΔW_a_/W_a_ initially averages around 48% for PI and 40% for PA6, and at two weeks post-treatment, it remains around 40% for PI and 30% for PA6. In comparison, previous results reported an initial ΔW_a_/W_a_ of 27–44%, which decreased to 24–28% after two weeks of ageing [15]. The relative enhancement is thus measurably better for the nitrogen-containing polymers studied here, particularly PI with the highest improvement in wettability and adhesion properties even after ageing. Table 5 provides a comparison of results across several classes of studied polymers [14,15], in terms of maximum adhesion work (W_a)_, maximum relative variation (ΔW_a_/W_a_), and minimum contact angle (WCA) values observed after reaching post-treatment surface equilibrium.

The equilibrium values are situated within about 100–125 mJ/m^2^, and are larger for the polar polymers (PET, PEEK, PMMA, PI, PA6) compared to nonpolar polymers (PEs, PP, PS). The highest increase in W_a_ is observed for the nonpolar polymers, due to the adhesion work calculated using Equation (2), where higher values of the contact angle θ result in a greater variation in W_a_. Therefore, the modification of hydrophobic surfaces (WCA > 90°), which evolve from hydrophobic to hydrophilic characteristics, appears more pronounced in terms of adhesion-related properties compared to the polar hydrophilic surfaces (WCA < 90°), which are in the hydrophilic domain both before and after surface processing [14,15].

The minimum WCAs are in an interval of about 6° for the nonpolar polymers, whereas the range covered for polar polymers is larger, about 12°, and PI shows the highest improvement in the wettability and adhesion-related properties.

**Figure 5:**
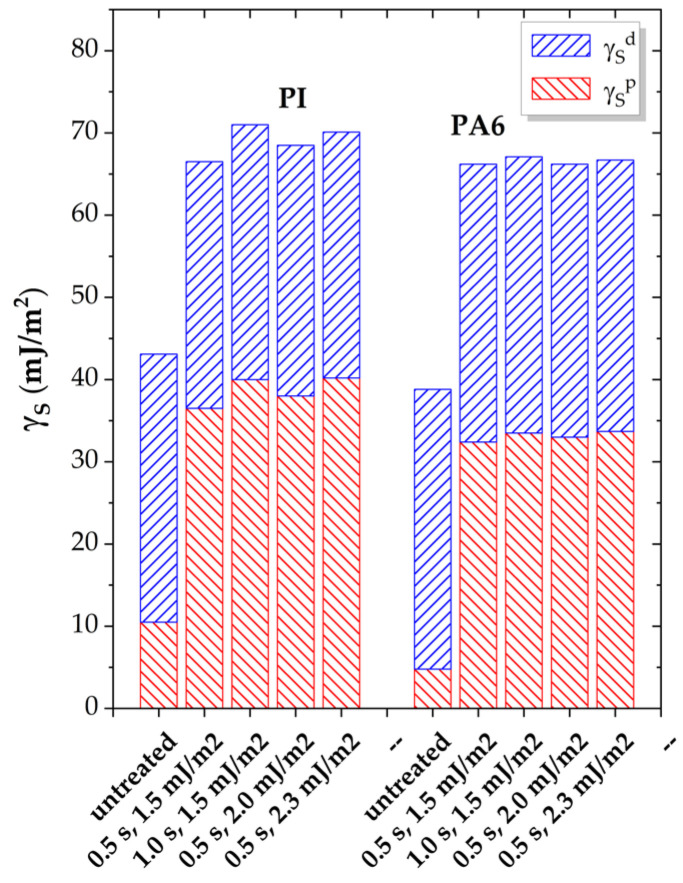
Polar (γ_S_^p^) and dispersive (γ_S_^p^) contributions to the surface energy (γ_S_) of PI and PA6 as a function of exposure time and DBD applied energy.

The calculation of the polar (γ_S_^p^) and dispersive (γ_S_^d^) components of the surface free energy (γ_S_), and by this means the surface polarity, using Equations (4) and (5) enables a correlation between the increase in wettability and adhesion work, on one hand, and the incorporation of the polar groups onto the surface on the other hand. These results are presented in Figure 5 and Table 6.

The two polymers, which contain oxygen- and nitrogen-bonded-to-carbon groups in their chain, exhibit measurable polarity before plasma treatment. Their dispersive components (γ_S_^d^) are similar, around 30 mJ/m^2^, while the polar components (γ_S_^p^) differ by a factor of approximately 2, with about 10 mJ/m^2^ for PI and around 5 mJ/m^2^ for PA6. These values agree with other findings in the literature [24,42,43,47,49,58,59]. This leads to a higher initial surface energy and twice the surface polarity of PI compared to PA6.

Upon plasma exposure, γ_S_^d^ remains unchanged within error margins, whereas γ_S_^p^ consistently increases up to about 40 mJ/m^2^ for PI and around 33 mJ/m^2^ for PA6. As a result, the total surface energy γ_S_ increases, falling within the range of approximately 67–70 mJ/m^2^ for both polymers. The surface polarity reaches a maximum of 0.57 for PI and 0.50 for PA6 (Table 6). The values are also very similar for the treatment parameters [1.0 s; 1.5 mJ] and [0.5 s; 2.0 mJ], indicating that higher applied DBD energy results in significant effects with shorter plasma exposures.

When comparing these results to those previously reported for other polar and nonpolar polymers, where surface polarity reached values of up to 0.60, the level of modification appears to be lower, especially for PI, which is more susceptible to surface modification in other respects [14,15]. This is due to the higher dispersive component of the surface energy (γ_S_^d^) of PI and PA6, resulting in a lower calculated γ_S_^p^/γ_S_ ratio according to Equation (5), despite the higher values of their polar component (γ_S_^p^).

### 3.3. Surface Chemical Structure

The oxygen content of the treated surfaces is measured to evaluate the influence of the two treatment parameters, exposure time and DBD applied power, on oxygen incorporation and the augmentation of the functionality of the PI and PA6 surfaces.

The two polymers exhibit structural functionalities characterized by carbon groups bonded to oxygen and nitrogen in an amide configuration (-N-C=O), with different chemical structures. PA6 has an aliphatic structure, while PI features a distinctive configuration in which all carbon atoms in its unit are bonded in cyclic groups (Figure 2). This structure, in principle, enhances the chemical stability of PI compared to other polymer classes and justifies its classification as a high-performance plastic. The initial oxygen content differs between the two polymers, with PI and PA6 containing 17.2 at. % O and 12.5 at. % O, respectively, levels comparable to previously studied PEEK. These values are notably lower than those found in PET and PMMA [15], which allows for the evaluation of factors influencing additional surface oxidation, specifically the stability of the chemical structure and/or the initial oxygen content.

Oxygen incorporation into the polymer structures is assessed using both the XPS survey spectra, which reveal the carbon, oxygen, and nitrogen components of the samples, and the high-resolution XPS C1s spectra.

The data obtained from the survey spectra are summarized in Table 7, presenting the total oxygen and nitrogen content as O1s/C1s and N1s/C1s ratios, respectively. These results demonstrate the progressive surface oxidation of both tested polymers, with the most significant modification occurring within the first 0.5 s of exposure. The oxygen content increases significantly in both polymers, with further, though less pronounced, increases observed with prolonged treatment. The variation as a function of discharge energy is significantly greater than that observed with exposure duration. The treatment’s effect is rapid, with modifications largely stabilized after the initial 0.5 s of exposure. This effect is determined by the number of reactive species generated in the plasma, which increases with the discharge applied energy. Based on the observed trends, exposure conditions of [0.5 s; 2.0 mJ] are optimal for effective surface functionalization.

The relative oxygen content is higher for plasma-treated PI compared to PA6, likely due to PI’s higher initial oxygen content. The total oxygen content on the treated PI surface reaches 22 at. % O, surpassing the maximum value of 18 at. % O observed for PA6. The APP treatment, even with short exposure times, enables additional oxidation in polymers like PI, which contain intrinsically bound oxygen in a cyclic repeat unit structure and are typically characterized by high chemical stability.

No measurable nitrogen incorporation is observed, as is typically the case with air plasma treatment. Incorporation of nitrogen during gas discharges exposure requires a nitrogen-rich gaseous environment, as N_2_ is a highly stable molecule that demands significant energy for dissociation, thereby limiting the generation of reactive nitrogen species in the plasma. Furthermore, the PI and PA6 polymer structures retain their nitrogen content after plasma treatment, maintaining it at a constant level.

These results are confirmed by the deconvolution of high-resolution XPS C1s spectra, fitted using reference measurements. Carbon groups are identified and numbered in ascending order of their binding energy (BE) [3,13,60], as shown in Table 8. Calibration of the BE scale is performed relative to the position of the hydrocarbon C1 (-C-C-, -C-H) core level, which exhibits a lower BE in PI (aromatic, cyclic) compared to PA6 (aliphatic). Beyond this, all other carbon groups are observed to have similar BE, within typical error margins (±2 eV).

The untreated PI and PA6 samples feature C1s spectra fitted with three components, positioned at similar BE for both materials, as detailed in Table 8. These components correspond to hydrocarbon atoms (C1), carbons singly bonded to a nitrogen atom (-C-N-) and/or oxygen atom (-C-O-) (C2), and carbons in amide groups (-N-C=O) (C3).

It is important to note that the BEs of carbons singly bonded to nitrogen and oxygen are very similar. As a result, their distinction becomes unreliable in the deconvolution of XPS C1s spectra, particularly after plasma exposure, which introduces new carbon-bonded-to-oxygen groups. The covalent bonds in polymer carbon species produce broader peaks compared to other materials, with full width at half maximum (FWHM) values of approximately 1.4–1.6 eV, leading to strong overlap within the C1s envelope. As a result, the separation of functional groups that differ by less than 1 eV in binding energy may be subject to considerable error.

PI displays a fourth characteristic peak at around 291.8 eV (C5), attributed to low-energy *π*–π* shake-up transitions accompanying the core-level ionization of aromatic carbons. For PI, the C1 peak exclusively represents aryl carbons, as all 22 carbons in its repeat unit are bonded in cyclic groups. The C1 peak in PA6 represents only aliphatic carbons.

**Figure 6:**
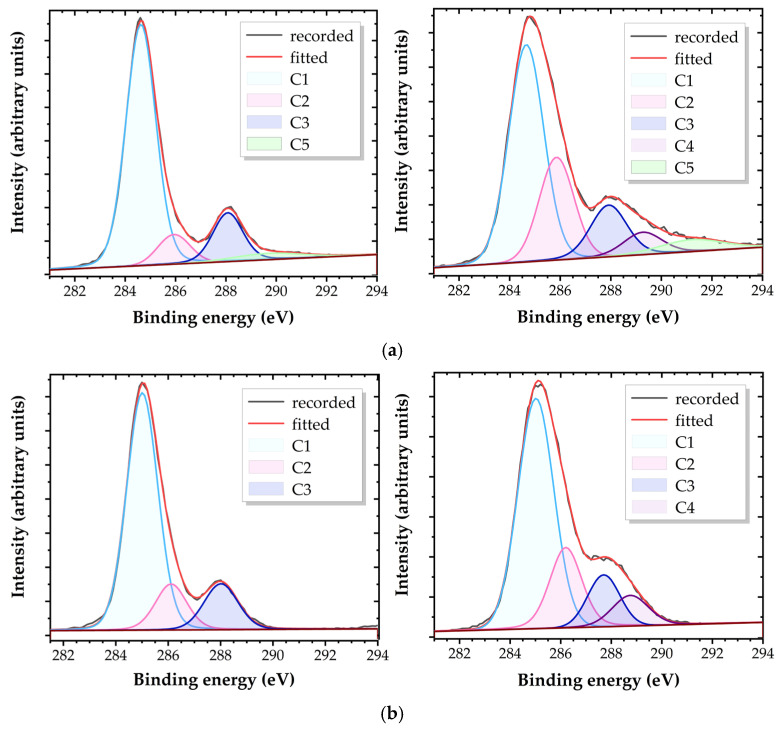
Typical deconvolutions of the high-resolution C1 XPS spectra for PI (**a**) and PA6 (**b**), untreated (**left**) and [0.5 s; 2.0 mJ] plasma-treated (**right**).

Plasma exposure induces the formation of new oxygen-bonded groups and/or enhances existing ones. The intensities of C2 and C3 components increase, and a new component assigned to carboxyl groups (-O-C=O) (C4) is observed, as shown in Figure 6.

Table 9 provides data on the relative atomic composition of carbon groups obtained from the deconvolution of high-resolution XPS C1s spectra, highlighting the presence of additional oxidized species on the plasma-treated surfaces.

The oxygen uptake can be calculated from these data, where the C2 component encompasses both -C-O- and -C-N- bonds, and C3 includes both carbonyl (–C=O) and amide (–N–C=O) bonds, while C4 exclusively represents carboxyl (–O–C=O) groups. Assuming no additional nitrogen is incorporated into the polymer structure, any observed increase in the intensities of C2, C3, and C4 can be attributed to the addition of oxygen.

Accordingly, Table 9 also presents the oxygen uptake (ΔO), calculated as

ΔO = ΔC2 + ΔC3 + ΔC4 = (C2_(treated)_ − C2_(untreated)_) + (C3_(treated)_ − C3_(untreated)_) + (C4_(treated)_ − C4_(untreated)_). (6)

These data broadly align with those presented in Table 7. The values derived from the C1s spectra are somewhat lower than those obtained from the survey spectra, likely due to the overlapping of peaks from certain functional groups, as well as differences in energy resolution during the recording of general spectra (1 eV energy step) versus high-resolution spectra (0.1 eV energy step). Nonetheless, the maximum level of oxygen incorporation remains higher for PI compared to PA6, and also surpasses that of other studied polar polymers [15]. This highlights the distinctive behaviour of the PI polymer, which shows a strong susceptibility to plasma-induced surface modifications.

The data in Table 7 and Table 9 suggest that maintaining a plasma treatment time of 0.5 s while varying the DBD applied energy offers a practical approach to tuning treatment outcomes.

The trends observed in the CA and XPS data differ to some extent. While the contact angle shows minimal variation, oxidation continues to increase with extended exposure time and higher applied energy beyond 0.5 s and 1.5 mJ/pulse, respectively. This behaviour occurs because CAMs primarily probe the outermost monolayer at the surface, whereas XPS explores about 50 Å at the usual 45° take-off angle. This indicates that hydrophilic modification at the surface levels out rapidly with plasma exposure, whereas functionalization progresses to some degree within the subsurface layers.

The data on surface properties related to oxygen uptake (Table 9), surface adhesion (Table 4), and polarity (Table 6) are in agreement, supporting the formation of new polar groups and the enhancement of existing ones on the surface. The additional carbon-bonded-to-oxygen groups (C2, C3, and C4) present on the surface are hydrophilic and exhibit significant polarity. This results in a marked increase in the surface’s hydrophilic character, thereby improving adhesion properties. The formation of oxidized functionalities aligns with the observed increase in the polar component of the surface energy, hence with the higher surface polarity. These results support the conclusion that effective surface functionalization has been achieved under the current treatment conditions.

### 3.4. Surface Morphology

AFM images show no significant physical changes to the surfaces, with both polymers displaying similar morphologies across all treatment parameters tested. The treatment does not cause surface degradation, even at higher discharge energy levels, likely due to the subsecond exposure.

**Figure 7:**
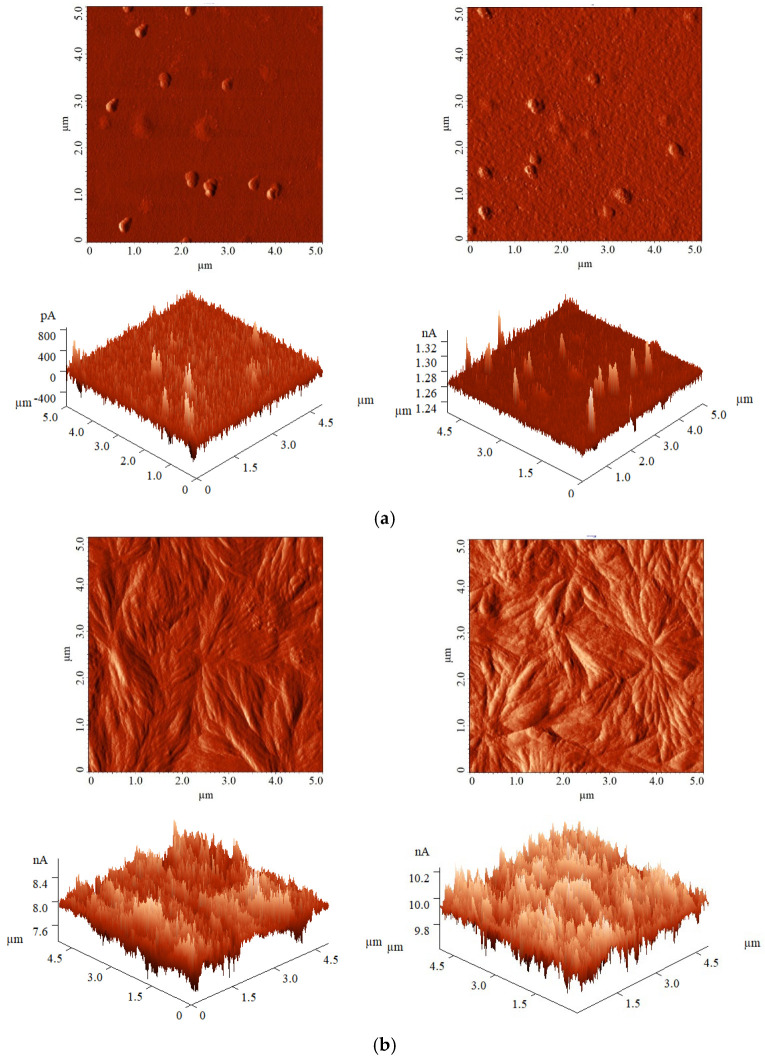
AFM images (5 μm × 5 μm) for PI (**a**) and PA6 (**b**), untreated (**left**) and [0.5 s; 2.0 mJ] plasma-treated (**right**).

Figure 7 presents examples of the surface topography of both polymers, for untreated and treated samples. The texture parameters related to surface morphology are presented in Table 10.

The two polymer materials exhibit different textures prior to treatment, with roughness values differing by approximately an order of magnitude. Plasma exposure results in minor changes, with the surfaces of PI and PA6 becoming slightly more textured, leading to an increase in roughness values of 1–3 nm. This result aligns with findings from other studies conducted under similar experimental setups [14] as well as in other DBD-type configurations at atmospheric pressure [61,62], where changes in average surface roughness were limited to only a few nanometers.

When considering the relative change in roughness compared to the untreated surfaces, the texturing of the PI surface is significantly more pronounced than that of the PA6 surface. The roughness of PI increases by a factor of approximately 2, representing practically a 100% increase, while the roughness of PA6 shows an average increase of only 25%. This behaviour can be attributed to the differences in the amorphous–crystalline structures of the two polymers. In the semicrystalline structure of PI, ablation occurs at a higher rate in the amorphous phase than in the crystalline phase, resulting in a pronounced texturing. In contrast, in the amorphous structure of PA6, ablation occurs uniformly throughout the material, leading to a reduced texturing effect.

The change in roughness depends on both treatment parameters, increasing with longer plasma exposure times and higher DBD energy. This behaviour differs from that of other surface properties, which typically exhibit less sensitivity to these treatment parameters. This difference arises because surface parameters measured by CA and XPS analyses are primarily determined by surface chemistry, while roughness is influenced by the physical changes associated with material removal from the polymer surface.

## 4. Conclusions

APP exposure significantly enhances the wetting and adhesion properties of nitrogen-containing polymers, with PI showing the most substantial improvements compared to PA6 and other studied polar polymers, even after ageing. PI exhibits pronounced texturing, a result of its semicrystalline structure. Additional oxidation occurs with very short exposure times. Increasing the energy applied to the discharge, while maintaining the plasma exposure duration at 0.5 s, optimizes the extent of surface modification. Under the treatment conditions used in this study, the primary mechanism of surface modification is functionalization, with no evidence of degradation observed.

## Figures and Tables

**Table 1:** Overview of the APP treatment parameters and results on polyimides and polyamides.

Material, Reference	Treatment Conditions	WCA	Recovery	Chemical Modification	Morphology, Mechanical Properties, Other
PI film [19]	APP torch, 400–1000 W, unspecified duration	61° → min. 28°	~50°, decrease in O/C values and peeling force over 30 days	O/C: 0.2 → max. 0.39; N/C: 0.09 → max. 0.17 (XPS)	strong etching effect; non-monotonous evolution of the peel force vs. power
PI film [21]	microAPP jet, Ar/H_2_O, 6–15 W, 60 s	70° → 25°	62° within 9 days	O/C: ~0.35 →~0.5: N/C: ~constant (XPS)	2.3 nm → 4.1 nm (AFM)
PI film [24]	DBD, Ar/O_2_, 100 W, 1 min	65° → 25°	-	increased oxygen content (XPS)	increased peel strength
PI (Kapton tape) [28]	He-0.15% H_2_O plasma jet, 5.2 W, 30 s	60° → 15°	-	O/C: 0.3 → 0.6; N/C: 0.06 → 0.1 (XPS)	non-uniform node-like structure R_a_: 11 nm → 41 nm
PI sheet [29]	APP, air, 30–120 s	polar surface energy doubles	-	-	150 nm → 250 nm (AFM), ablation (SEM), increased adhesive bond strength
PI membranes [30]	APP air, 150 W, 2–10 min	56° → 14° → superhydrophilic	~14° over unspecified interval	C=O and -OH groups (FTIR)	streaks on SEM images; slight surface damage; minor changes in peel force
PI powder particles [31]	DCSBD, air, 400 W, 5 min	-	-	O/C: 0.19 → ~0.29; N/C: constant (XPS)	improved dispersability in water, embedding in metal layers, wear behaviour
PI film [32]	microwave line APP, Ar/O2, 3.5 kW, 0–100 ms	70° → min. 18°	-	-	rapid ashing
PI film [33]	microwave APP jet, Ar, 40 W, 1–5 min	66° → 24° → 8°	-	-	etching, nanoscale particles
PI (Kapton films) [34]	DBD, air, 30 W, 1–20 s (40 times higher to swipe the samples)	80° → 24°	~60° over extended interval (>1 month)	O/C: 0.2 → ~0.3; N/C: constant (XPS)	randomly distributed nanostructures, S_q_: 1 nm → 25 nm due to fast etching (4 nm/s)
PI film [35]	APP jet, air, 300–600 W, unspecified duration	85° → ~30°	-	small increase in C=O and C-O groups (XPS)	decrease in tensile strength, increase in peel strength attributed to etching
PI film [36]	APP, Ar, 100 W, unspecified duration	68° → 25°	-	reduced chemical modification (XPS)	improved coating uniformity
PI/PES membranes [37]	APP jet (60 W) and corona (25 kV), unspecified duration	-	-	-	altered fibre morphology, improved filter efficiency by corona
PI film [38]	APP, O_2_, N_2_, unspecified parameters	-	-	O/C: 0.13 → 0.32 (XPS)	high-temp PI: 4 nm → 6 nm, 12 nm; low-temp PI: 0.1 nm → ~1 nm (SEM); increased shear force
PI [39]	DBD, He, He-O2, 200–300 W RF, 20–60 s	82° → 7°	complete hydrophobic recovery within 7 days	increase in -C=O and -OH groups (FTIR)	cluster formation, 0.9 nm → 6.8 nm (AFM)
PI film [40]	RF plasma, O_2_, 2 kPa, 10 min	75° → 26°	-	O/C: 0.2 → 0.3; N/C: ~constant (XPS)	unchanged roughness (AFM)
PI film [41]	DBD, Ar, 5.4 W, 0–300 s	78° → 10–20°	-	some indication on oxidation (FTIR)	strong morphology modification at micrometre scale (SEM)
PI film [42]	APGD, water cathode, air, 5–90 s	68° → 46°	62° within 11 days	-	6 nm → 13 nm (AFM)
PI film [43]	APP, RF, Ar-O_2_, 0–300 W, 1 min	increased polar surface energy	ageing within 7 days	O/C: 0.23 → max. 0.39 (XPS)	increased peel strength
PA6 [27]	DCSBD, air, 320 W, 30 s	70° → 28°	continued recovery for >2 months	O/C: 0.17 → 0.3 N/C: constant (XPS)	smoother surface at micrometric level; sample heating
PA12 [44]	microwave surface wave jet, Ar (+O_2_, N_2_), ~1 cm^2^ exposed area, 25 ms^−1^ s	79° → min. 19°	~50° within 30 days	O/C: 0.3 → 0.6 N/C: 0.05 → 0.09 (XPS)	26 nm → 90 nm (AFM)
PA6 foils [45]	VDBD and DCSBD, hundreds of W, 0.25–2 s	DCSBD: 65° → 41° → 28° VDBD: 65° → 47° → 45°	DCSBD: >40°; VDBD: >55° within 14 days	O/C: 0.2 → max. 0.4; N/C: ~constant (XPS)	peel resistance increases beyond error bars for at least 1 s exposure
PA66 [46]	DBD, air, 0.5–32 s	64° → 30° (0.5 s) → 25°	30–35° within 4 days	O/C: 0.2 → 0.6; N content constant (XPS)	14 nm → 35 nm (AFM)
PA6, PA12 moulded sheets [47]	microdischarge, 24-needle electrode system, unspecified duration	PA6: 76° → 26°; PA12: 82° → 40°	PA6: >50° PA12: >60° within 2–3 h	-OH, C=O and C-O bands, decreasing over ageing (FTIR)	S_q_: PA6: 21→24 nm PA12: 10→12 nm
PA11, PA12 sheets [48]	APP torch, air, unspecified duration	PA11: 93° → 26°; PA12: 83° → 22°	complete recovery within 21 days	PA11: O/C: 0.4 → 0.9; N/C: 0.2 → 0.4 (XPS)	decrease in roughness parameters
PA66 [49]	DCSBD, air, 320 W, 30 s	63° → 33°	~55° within 8 days and continued >2 months	O/C: 0.16 → 0.47; N/C: 0.15 → 0.23 (XPS)	smoother surface at micrometric level; sample heating

**Table 2:** Surface tension components of test liquids used for contact angle measurement.

Test Liquid	γ_L_ (mJ/m^2^)	γ_L_^d^ (mJ/m^2^)	γ_L_^p^ (mJ/m^2^)
Water (W)	72.8	21.8	51.0
Formamide (F)	58.2	35.1	23.1

**Table 3:** Contact angle of water (WCA) (°) for PI and PA6 on the untreated and plasma-treated surfaces as a function of exposure time and DBD applied energy.

Time (s)	Untreated	0.5	1.0	0.5	0.5
E_DBD_ (mJ/Pulse)		1.5	1.5	2.0	2.3
**PI**	78.7 ± 1.1	42.6 ± 0.8	35.9 ± 0.7	38.0 ± 0.8	35.6 ± 0.8
**PA6**	75.9 ± 1.0	45.7 ± 0.9	39.6 ± 0.8	41.5 ± 0.9	39.5 ± 0.8

**Table 4:** Adhesion work and relative variation in the adhesion work calculated for PI and PA6 on the untreated surfaces, plasma-treated surfaces, and aged (14 days) plasma-treated surfaces as a function of exposure time and DBD applied energy.

	PI					PA6				
Time (s)	Untreated	0.5	1.0	0.5	0.5	Untreated	0.5	1.0	0.5	0.5
E_DBD_ (mJ/Pulse)		1.5	1.5	2.0	2.3		1.5	1.5	2.0	2.3
**W_a_ (mJ/m^2^)**	87.1	126.4	129.5	130.2	132.0	90.5	123.6	128.9	127.3	129.0
**ΔW_a_/W_a_ (%)**	—	45%	49%	49%	51%	—	37%	42%	41%	42%
**ΔW_a_/W_a_ (%) (aged)**	—	40%	43%	37%	37%	—	30%	32%	30%	28%

**Table 5:** Maximum values of the adhesion work and its relative variation and minimum values of water contact angle on 14 days post-treatment aged polymer surfaces.

	PEs, PP	PS	PET	PEEK	PMMA	PI	PA6
	[14]	[14]	[15]	[15]	[15]		
**W_a_ (mJ/m^2^)**	103	113	117	120	112	124	119
**ΔW_a_/W_a_ (%)**	80%	65%	27%	27%	22%	43%	32%
**WCA (°)**	62	56	53	49	57	45	50

**Table 6:** Surface polarity of PI and PA6 as a function of exposure time and DBD applied energy.

γ_S_^p^/γ_S_	Time (s)	Untreated	0.5	1.0	0.5	0.5
	E_DBD_ (mJ/Pulse)		1.5	1.5	2.0	2.3
**PI**		0.243	0.548	0.563	0.554	0.573
**PA6**		0.123	0.489	0.499	0.498	0.505

**Table 7:** Relative atomic concentrations (at. %, ±1 at. %) and surface composition of PI and PA6 as a function of exposure time and DBD applied energy.

	PI					PA6				
Time (s)	Untreated	0.5	1.0	0.5	0.5	Untreated	0.5	1.0	0.5	0.5
E_DBD_ (mJ/Pulse)		1.5	1.5	2.0	2.3		1.5	1.5	2.0	2.3
C1s	80	77	75	74	72	81	75	73	73	72
O1s	13	16	18	20	22	10	15	16	17	18
N1s	7	7	7	6	6	9	10	11	10	10
O1s/C1s	0.16	0.21	0.24	0.27	0.30	0.12	0.20	0.22	0.23	0.25
N1s/C1s	0.09	0.09	0.09	0.08	0.08	0.11	0.13	0.15	0.14	0.14

**Table 8:** Binding energies of carbon functional groups in the C1s fitted spectra for PI and PA6 (±2 eV).

Functional Groups	Assignment	BE (eV)
carbon/hydrogen -C-C-, -C-H	C1	284.6, 285.0
carbon/nitrogen or oxygen -C-N-, -C-O-	C2	286.0, 286.5
amide –N–C=O	C3	288.0
carboxyl –O–C=O	C4	289.0
*π*–π* shake-up	C5	~291.8

**Table 9:** Atomic composition of the carbon species C1s (in at. %, ± 1 at. %) and oxygen uptake (ΔO, in at. %, ± 1 at. %) for PI and PA6 as a function of exposure time and DBD applied energy.

	PI					PA6				
Time (s)	Untreated	0.5	1.0	0.5	0.5	Untreated	0.5	1.0	0.5	0.5
E_DBD_ (mJ/Pulse)		1.5	1.5	2.0	2.3		1.5	1.5	2.0	2.3
C1	73	59	55	54	51	70	63	61	59	56
C2	11	16	17	19	21	14	17	18	20	22
C3	12	13	13	11	12	15	14	14	13	14
C4	-	8	10	12	11	-	6	7	8	8
C5	4	4	5	4	5	-	-	-	-	-
ΔO	—	14	18	19	22	—	7	9	11	14

**Table 10:** Roughness values S_a_, S_q_, and S_y_ (in nm, ±0.5 nm) for PI and PA6 as a function of exposure time and DBD applied energy.

	PI					PA6				
Time (s)	Untreated	0.5	1.0	0.5	0.5	Untreated	0.5	1.0	0.5	0.5
E_DBD_ (mJ/Pulse)		1.5	1.5	2.0	2.3		1.5	1.5	2.0	2.3
**S_a_**	2.2	3.5	4.7	4.9	5.1	9.3	10.2	10.8	11.1	11.4
**S_q_**	3.1	4.8	5.9	6.2	6.5	11.9	13.2	13.5	14.0	14.5
**S_y_**	33	45	64	69	73	95	129	138	143	147

## Data Availability

Data are contained within the article.
